# Estimated Burden of Keratitis — United States, 2010

**Published:** 2014-11-14

**Authors:** Sarah A. Collier, Michael P. Gronostaj, Amanda K. MacGurn, Jennifer R. Cope, Kate L. Awsumb, Jonathan S. Yoder, Michael J. Beach

**Affiliations:** 1 Division of Foodborne, Waterborne, and Environmental Diseases, National Center for Emerging and Zoonotic Infectious Diseases, CDC

Keratitis, inflammation of the cornea, can result in partial or total loss of vision and can result from infectious agents (e.g., microbes including bacteria, fungi, amebae, and viruses) or from noninfectious causes (e.g., eye trauma, chemical exposure, and ultraviolet exposure). Contact lens wear is the major risk factor for microbial keratitis (1–3); outbreaks of *Fusarium* and *Acanthamoeba* keratitis have been associated with contact lens multipurpose solution use (4,5), and poor contact lens hygiene is a major risk factor for a spectrum of eye complications, including microbial keratitis and other contact lens–related inflammation (3,6,7). However, the overall burden and the epidemiology of keratitis in the United States have not been well described. To estimate the incidence and cost of keratitis, national ambulatory-care and emergency department databases were analyzed. The results of this analysis showed that an estimated 930,000 doctor’s office and outpatient clinic visits and 58,000 emergency department visits for keratitis or contact lens disorders occur annually; 76.5% of keratitis visits result in antimicrobial prescriptions. Episodes of keratitis and contact lens disorders cost an estimated $175 million in direct health care expenditures, including $58 million for Medicare patients and $12 million for Medicaid patients each year. Office and outpatient clinic visits occupied over 250,000 hours of clinician time annually. Developing effective prevention messages that are disseminated to contact lens users and investigation of additional preventive efforts are important measures to reduce the national incidence of microbial keratitis.

Because a specific *International Classification of Diseases, Ninth Revision, Clinical Modification* (ICD-9-CM) code for microbial keratitis does not exist, a set of keratitis-related codes that could apply to microbial keratitis patients was developed with clinician input. Codes included corneal ulcer (370.0), other forms of keratitis resulting from an underlying condition (370.8 used in conjunction with a second diagnostic code for the underlying condition [e.g., *Acanthamoeba* or *Fusarium* infection]), unspecified keratitis (370.9), and corneal disorders involving contact lens use (371.82). For office and outpatient visits, “contact lens problems” as a reason for the visit also were included in the contact lens category. The 2010 Marketscan Commercial Claims and Encounters database* was used to characterize the number of visits per person and episode length of keratitis and contact lens related–problems in 2010. The Marketscan Commercial, Medicare, and Medicaid databases also were used to estimate costs per visit for office, outpatient, or emergency department visits that did not result in hospital admission for keratitis, by insurance source. Data from the 2006–2010 National Ambulatory Medical Care Survey (NAMCS), National Hospital Ambulatory Care Medical Survey of Outpatient Departments (NHAMCS-OPD),† and 2010 Nationwide Emergency Department Sample§ were used to generate annual estimates of office, outpatient, and emergency department visits for the ICD-9-CM codes of interest. Statistical software was used to apply sampling weights and account for the complex sample design of these surveys. To estimate the total cost of annual visits, the total number of annual visits was multiplied by the cost per visit.

In 2010, the mean cost of a visit to a doctor’s office for a keratitis-related diagnostic code was $151, and the mean cost of an emergency department visit was $587 (Table 1). Most patients in 2010 made only a single visit, but a small proportion had numerous follow-up visits (maximum 49 total visits). Based on NAMCS and NHAMCS-OPD data, an estimated 700,000 doctor’s office and outpatient clinic visits for keratitis occurred in 2010, including 280,000 visits for corneal ulcers (Table 2). The majority of visits (76.5%) were associated with antimicrobial prescriptions. Separately, an estimated 230,000 doctor’s office and outpatient clinic visits for corneal disorders involving contact lenses occurred, with the majority (70.0%) resulting in antimicrobial prescriptions. Among emergency department visits, 19,000 visits for corneal disorders involving contact lenses and 41,000 visits for keratitis occurred in 2010, including 25,000 visits for corneal ulcers. Approximately 1% of office visits and 4% of emergency department visits involved both categories of diagnosis codes. Women made 63.3% of office visits and 54.7% of emergency department visits. Persons aged <25 years made 20.5% of all visits, persons aged 25–44 years made 29.2% of visits, persons aged 45–64 years made 25.3% of visits, and persons aged ≥65 years made 25.1% of visits.

The total cost of the estimated 988,000 visits to doctor’s offices, outpatient clinics, and emergency departments for keratitis and contact lens related diagnostic codes was $174.9 million, including $58.0 million in costs for Medicare patients and $11.9 million in costs for Medicaid patients (Table 3). Office and outpatient clinic visits occupied over 250,000 hours of clinician time annually.

## Discussion

Nearly 1 million clinical visits for keratitis occur annually. The largest single risk factor for microbial keratitis is contact lens wear (1). Among the estimated 38 million contact lens wearers in the United States (8), poor storage case hygiene, infrequent storage case replacement, and overnight lens wear are established preventable risk factors for microbial keratitis, contact lens–related inflammation, and other eye complications (3,6,7).

In this analysis, the proportion of visits varied by age and sex. This likely reflects differences in contact lens use and inclination toward seeking health care as well as differences in risk factors for keratitis. The incidence of microbial keratitis reported previously ranged from 0.4 to 5.2 per 10,000 person-years for rigid gas-permeable and soft contact lens wearers to >20 per 10,000 person-years for overnight soft contact lens wearers (9); one population-based study in California estimated that 71,000 cases of severe microbial keratitis could occur per year (10). This study is the first attempt to characterize the overall burden of keratitis on the U.S. health care system. To help direct future prevention efforts for microbial keratitis, the current epidemiology of keratitis in the United States and its impact on the U.S. health care system must be understood and quantified. Additionally, development and dissemination of effective prevention messages to contact lens users is essential.

The findings in this report are subject to at least four limitations. First, the estimated prevalence of visits for keratitis-related diagnostic codes is likely to be an underestimate, because the datasets used in this analysis capture few visits to optometrists. Although most persons with sight-threatening cases of microbial keratitis would be expected to visit an ophthalmologist, persons with less complicated infections might only interact with an optometrist, and those visits would not be included in the datasets used. Second, not all keratitis visits were for microbial keratitis; some keratitis does not result from infection. Many ICD-9-CM codes for keratitis identify keratitis by anatomic location (e.g., central corneal ulcer compared with marginal corneal ulcer) rather than by etiologic agent; therefore, visits that involved microbial keratitis could not be specifically identified. Although a large percentage of patients received antimicrobial treatment, and ICD-9-CM codes specific for noninfectious keratitis were excluded from the analysis, the proportion of keratitis caused by infectious agents is unknown. The percentage of patients receiving antimicrobial treatment is likely an underestimate because Marketscan does not record prescriptions not covered by insurance (i.e., compounded prescriptions or prescriptions that cost less than the copay amount). Third, this analysis was not able to directly identify contact lens wearers. Some visits for keratitis likely occurred among persons who do not wear contact lenses, but that proportion is unknown. Conversely, visits for corneal disorders involving contact lens wear are not all caused by microbial keratitis (e.g. an unknown proportion were caused by corneal abrasions), although the majority received topical antimicrobials. Finally, because the demographics of contact lens wearers in the United States are not known, rates of visits by age or sex among contact lens wearers could not be calculated.

Keratitis associated with poor contact lens hygiene is preventable. Prevention efforts should include surveillance, improved estimates of the burden of disease, and vigorous health promotion activities focused on contact lens users and eye care professionals (ophthalmologists, optometrists, and opticians). Increased surveillance capacity is needed for microbial keratitis, in particular data from optometrist visits. Current recommendations for proper contact lens wear and care are available (Box).¶

BOX Recommendations for contact lens wear and care to reduce the risk for microbial keratitis*

**Contact lens habits and hygiene**

- Wash hands with soap and water. Dry hands well with a clean cloth before touching contact lenses each time they are inserted or removed.
- Don’t sleep in contact lenses unless prescribed to do so by an eye care provider.
- Keep water away from contact lenses. Avoid showering while wearing contact lenses, and remove them before using a hot tub or swimming.

**Contact lenses and supplies**

- Rub and rinse contact lenses with contact lens disinfecting solution, never water or saliva, to clean them each time they are removed.
- Never store contact lenses in water.
- Replace contact lenses as often as recommended by an eye care provider.
- Rub and rinse contact lens case with contact lens solution, never water, and then empty and dry with a clean tissue. Store upside down with the caps off after each use.
- Replace contact lens case at least once every 3 months.
- Don’t “top off” solution. Use only fresh contact lens solution in the case. Never mix fresh solution with old or used solution.
- Use only the contact lens solution recommended by an eye care provider.

**Eye care provider involvement**

- Visit an eye care provider yearly or as often as recommended by your primary health care provider.
- Ask an eye care provider about how to care for contact lenses and supplies.
- Remove contact lenses immediately and call an eye care provider if experiencing eye pain, discomfort, redness, or blurred vision.
- Carry a backup pair of glasses with a current prescription in case contact lenses need to be removed.

Additional information about healthy contact lens wear and care is available at http://www.cdc.gov/contactlenses and http://www.cdc.gov/contactlenses/show-me-the-science.html.

What is already known on this topic?

Microbial keratitis is an infection of the cornea caused by bacteria, fungi, amebae, or viruses and can result in vision loss or blindness. Improper contact lens wear, particularly poor storage case hygiene, infrequent storage case replacement, and overnight lens wear, is the largest risk factor for microbial keratitis.

What is added by this report?

Each year, an estimated 930,000 doctor’s office and outpatient clinic visits and 58,000 emergency department visits for keratitis and conditions caused by contact lens wear cost $175 million in direct health care expenditures and occupy over 250,000 hours of clinician time.

What are the implications for public health practice?

Increased surveillance capacity, identification of additional preventive measures, and further quantification of the burden of keratitis can help to direct prevention efforts. Development of effective prevention messages for dissemination to contact lens users and eye care professionals, including ophthalmologists and optometrists, is important to reduce microbial keratitis.

## Figures and Tables

**TABLE 1:** Estimated episode characteristics and cost of visits to doctor’s offices, outpatient clinics, and emergency departments involving keratitis-related diagnostic codes — United States, 2010*

			Cost per visit	
			
Diagnosis	Median no. of visits per episode (range)	Median episode length (range)	Doctor’s office/Outpatient clinic ($)	Emergency department ($)
Corneal disorder due to contact lens (ICD-9-CM code 371.82)	1 (1–10)	1 day (1–343)	104	505
Keratitis (370.0, 370.8, 370.9)	1 (1–49)	1 day (1–359)	157	622
Corneal ulcer (370.0)	2 (1–48)	3 days (1–359)	155	658
Other forms of keratitis (370.8)	1 (1–18)	1 day (1–345)	247	672
Unspecified keratitis (370.9)	1 (1–25)	1 day (1–359)	152	564
Any contact lens or keratitis related diagnosis (371.82, 370.0, 370.8, 370.9)	1 (1–49)	1 day (1–359)	151	587

**Abbreviation:** ICD-9-CM = *International Classification of Diseases, Ninth Revision, Clinical Modification.*

* **Source:** Marketscan Commercial Claims and Encounters and Medicare Supplemental and Multistate Medicaid databases.

**TABLE 2:** Estimated number of visits to doctor’s offices, outpatient clinics, and emergency departments for keratitis-related diagnostic codes — United States, 2010

Diagnosis	No. of doctor’s office/Outpatient clinic visits* (95% CI)	No. of emergency department visits† (95% CI)
Corneal disorder due to contact lens (ICD-9-CM code 371.82)	230,000 (130,000–340,000)	19,000 (14,000–25,000)
Keratitis (370.0, 370.8, 370.9)	700,000 (510,000–890,000)	41,000 (30,000–52,000)
Corneal ulcer (370.0)	280,000 (170,000–390,000)	25,000 (18,000–33,000)
Other forms of keratitis (370.8)	—§	7,000 (3,000–12,000)
Unspecified keratitis (370.9)	380,000 (250,000–520,000)	8,000 (7,000–9,000)
Any contact lens or keratitis related diagnosis (371.82, 370.0, 370.8, 370.9)	930,000 (690,000–1,170,000)	58,000 (43,000–72,000)

**Abbreviations:** ICD-9-CM = *International Classification of Diseases, Ninth Revision, Clinical Modification;* CI = confidence interval.

* **Source:** 2006–2010 National Ambulatory Medical Care Survey/National Hospital Ambulatory Care Medical Survey of Outpatient Departments.

† **Source:** 2010 Nationwide Emergency Department Sample.

§ Data not reported because of small sample size.

**TABLE 3:** Estimated total annual cost of visits in millions of dollars, by insurance source, to doctor’s offices, outpatient clinics, and emergency departments involving keratitis-related diagnostic codes — United States, 2010*

	Insurance source (millions of $)				
		
Diagnosis	Private	Medicare	Medicaid	Other†	Total
Corneal disorder due to contact lens (ICD-9-CM code 371.82)	21.5	3.9	4.6	3.6	**33.5**
Keratitis (370.0, 370.8, 370.9)	62.5	49.3	5.4	18.0	**135.3**
Any contact lens or keratitis related diagnosis (371.82, 370.0, 370.8, 370.9)§	83.8	58.0	11.9	21.2	**174.9**

**Abbreviation:** ICD-9-CM = *International Classification of Diseases, Ninth Revision, Clinical Modification.*

* **Sources:** 2006–2010 National Ambulatory Medical Care Survey/National Hospital Ambulatory Care Medical Survey of Outpatient Departments, 2010 Nationwide Emergency Department Sample, and 2010 Marketscan Commercial Claims and Encounters, Medicare Supplemental and Multistate Medicaid databases.

† Includes patients with other types of insurance (e.g., Tricare, the military health plan), uninsured patients, and patients with an unknown source of insurance.

§ The amount for any diagnosis does not equal the sum of visits with a corneal disorder caused by contact lens use diagnosis and visits with a keratitis diagnosis because approximately 1% of office visits and 4% of emergency department visits involved both categories of diagnosis codes.

## Footnotes

* The Marketscan Commercial Claims and Encounters, Medicare Supplemental, and Multistate Medicaid databases, from Truven Health analytics, include insurance claims and payments. Costs are the sum of 2010 insurer and out-of-pocket payments for office, outpatient, or emergency department visits per patient.

† CDC’s national sample of visits to nonfederally employed, office-based physicians (NAMCS) and outpatient departments of nonfederal, general, and short-stay hospitals (NHAMCS-OPD), from the National Center for Health Statistics. Multiple years of data were used to increase sample size. Data for 2010 are the most recent year available.

§ A national sample of hospital-based emergency department visits from the Healthcare Cost and Utilization Project, Agency for Healthcare Research and Quality.

¶ Available at http://www.cdc.gov/contactlenses.

* These recommendations were developed by CDC in collaboration with a workgroup that included members from the Food and Drug Administration, the American Academy of Ophthalmology, the American Academy of Optometry, the American Optometric Association, the Contact Lens Association of Ophthalmologists, the Contact Lens Society of America, and the National Academy of Opticianry.
